# Climate Change and Health Research: Time for Teamwork

**DOI:** 10.1289/ehp.12150

**Published:** 2008-11

**Authors:** Sharon H. Hrynkow

**Affiliations:** National Institute of Environmental Health Sciences, National Institutes of Health, Department of Health and Human Services, Bethesda, Maryland, E-mail: hrynkows@niehs.nih.gov

Commitments to address climate change, including those aimed at reducing greenhouse gases by 2050, are increasing. As efforts toward mitigation of climate change gather momentum, there is an increased need to understand the linkages between climate change and human health and the potential health consequences of mitigation strategies. Although there is substantial knowledge of how climate change can affect human health, there is much the scientific community does not know or understand.

McMichael et al. (2004) calculated that approximately 166,000 deaths per year and 5.5 million disability adjusted life years (a measure of healthy life years lost) resulted from climate change in the year 2000. These figures underestimate the scope of the problem for three reasons: *a*) They are based on only a few health end points for which global data are readily available; *b*) they do not fully capture the range of potential indirect impacts on human health; and *c*) they do not provide estimates of impact beyond 2000. The first order of business should be the development of metrics to understand the magnitude of climate change impacts on human health, reflecting the long-term consequences and the noncommunicable and indirect health impacts.

Development of priority research questions and gap areas in our knowledge base is another important issue. Although lists of health research priorities have been generated over the past decade, new technologies and realities require that we revisit these well-considered lists and determine whether we can identify new, practical research questions that will inform current policy-making efforts. In a workshop sponsored by the National Institute of Environmental Health Sciences on 16 April 2008 (Hrynkow 2008), science, health, and development experts were brought together to consider potential actions and activities related to climate change and human health. A number of underrecognized yet highly relevant themes emerged, all deserving consideration. For example, new modeling paradigms are needed to understand more completely how subtle changes in factors such as temperature and humidity influence the eventual exposure to humans to contaminants via air, water, and soil. We need to know how slight increases in temperature, humidity, or other weather features favor the emergence, reemergence, or redistribution of infectious agents, and if so, where these agents will emerge. It is also likely that risk factors for respiratory diseases such as allergy and asthma will change due to the increase in temperature and new dispersal patterns of allergens and air pollutants. Better tools to measure these changes and analyze existing environmental monitoring data are needed. Information from such studies could help develop risk maps that facilitate planning and preparedness, particularly as related to children and other vulnerable groups.

The global response to climate change will also require mitigation strategies involving the use of new energy sources. It will be important to determine if the widespread use of “greener” fuels will pose health challenges to workers who produce and distribute them, to the consumers that use them, and to the environment in general. Policy makers need to know which solutions carry co-benefits and have a high probability of facilitating adaptation to the changing climate. We must also be able to anticipate the health and environmental impacts of new energy strategies and avoid a legacy of unintended consequences.

The complex nature of the interaction between climate change and human health requires a multidisciplinary, integrated approach, which will most likely consist of efforts from a variety of regulatory, research, and public health agencies. The development of multidisciplinary research programs on global climate change and human health would not only provide benefits from the scientific standpoint but also from potential cost-sharing built into interagency collaborations. At a time of low-growth budget projections, such approaches are particularly attractive.

Finally, as new tools are developed to assist individuals and communities in adapting to climate change, a social and behavioral research agenda will be essential. Health economists can play a role as nations examine impacts of action and inaction in terms of public health. The scale-up of effective pilot programs, such as those that provide early warnings to communities during heat waves, will demand involvement of health practitioners, social scientists, and public policy officials, among others.

Human health will be an important backdrop upon which our social, economic, and environmental decisions related to climate change will be played. To protect public health from the impacts of climate change, we must develop a more comprehensive body of evidence to inform decision makers and policy makers. Developing this evidence base will require involvement of physicists, chemists, social scientists, environmental groups, environmental regulators, public health professionals, and health scientists, among others. Environmental health scientists and agencies must step up in new ways to develop critical knowledge on risks, develop and deploy effective strategies to reduce and prevent human suffering, and participate in the thorough analysis of energy options that have been suggested to mitigate climate change. With all of the work ahead of us, we need to begin now.

## Figures and Tables

**Figure:**
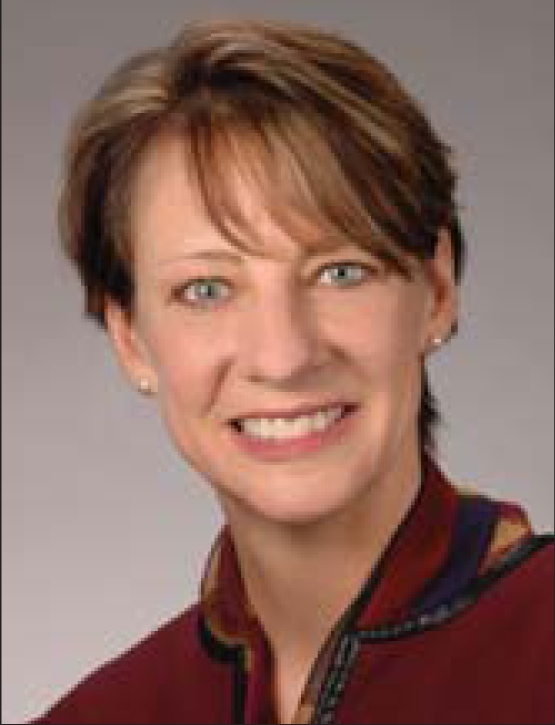
Sharon H. Hrynkow
